# Supplementary material for: When the Beetles Hit the Fan: The Fan-Trap, an Inexpensive, Light and Scalable Insect Trap under a Creative Commons License, for Monitoring and Experimental Use
Source: Insects. 2022 Dec 5;13(12):1122. doi: 10.3390/insects13121122 (PMC9784568; doi:10.3390/insects13121122)
Supplement: Supplementary file 1 [file insects-13-01122-s001.zip › Supplementary_S1_Fantrap_description.pdf]

S-1 – Practical information for constructing the *Fan-traps*.

Our own experience

The main part of the fan-trap, the "arrow part", was cut out of a polypropylene sheet (65 x 100 cm and 0.8 mm thick) using a Metaquip MQ1590 laser machine implemented with the RDWorks 8.0 system. The laser machine used has a working area of 150 x 90 cm, a laser power of 130 W, a cutting speed of up to 600 mm/sec with a positional accuracy of 0.01 mm.

To cut the 0.8 mm thick polypropylene sheet, the settings were: a speed of 60 mm/sec, a minimum and maximum power of 25 W and 50 W, respectively. The minimum power should always be half the maximum power to avoid burning the polypropylene when the laser is temporarily stationary, e.g., when cutting corners.

Previous tests have shown that a thicker sheet (maximum 1 mm) could work just as well with these blueprints, but the settings will need to be adjusted. Also, our personal observation has shown that lens wear could also affect the settings.

The costs involved (material and manpower) were as follows: 5,33 € for one polypropylene sheet (65 x 100 cm and 0.8 mm thick), out of which five traps were cut (cost per unit: 1.07 €). Fifteen sheets (= 75 *fan-traps*) could be processed within one hour. Counting a 30 €/h charge for manpower, the handling cost per trap was 0.4 €. The total cost per trap was thus 1.47 €, to which a modest FabLab fee (50 €/year) should be added, as well as the time for setting up the laser cutter each day (15 min, 7.5 €). So, each of the *fan-traps* we produced cost less than 2 €.

Possibilities for changes

The design can be easily modified with any conventional drawing software. In the present case, the open-source software, Inkscape 1.1 (https://inkscape.org/) was used. Figure 1 shows all the measurements of the fan-trap, but the scale is maintained in the blueprint files.

Three types of drawings are included here, one without fixation holes (Fantrap1.svg) and two with various fixations (Fantrap2.svg; Fantrap3.svg). To maximise trap production, an open-source nesting software such as Deepnest (https://deepnest.io/) can be used (see Fantrap3nested.ai). The software will set as many traps as possible according to the size of the plate and merge the contact line to facilitate cutting. The drawings are in .svg or .ai format, which should be readable by most laser cutters. If a machine cannot read the files, it is possible to convert them using Inkscape or another drawing software.

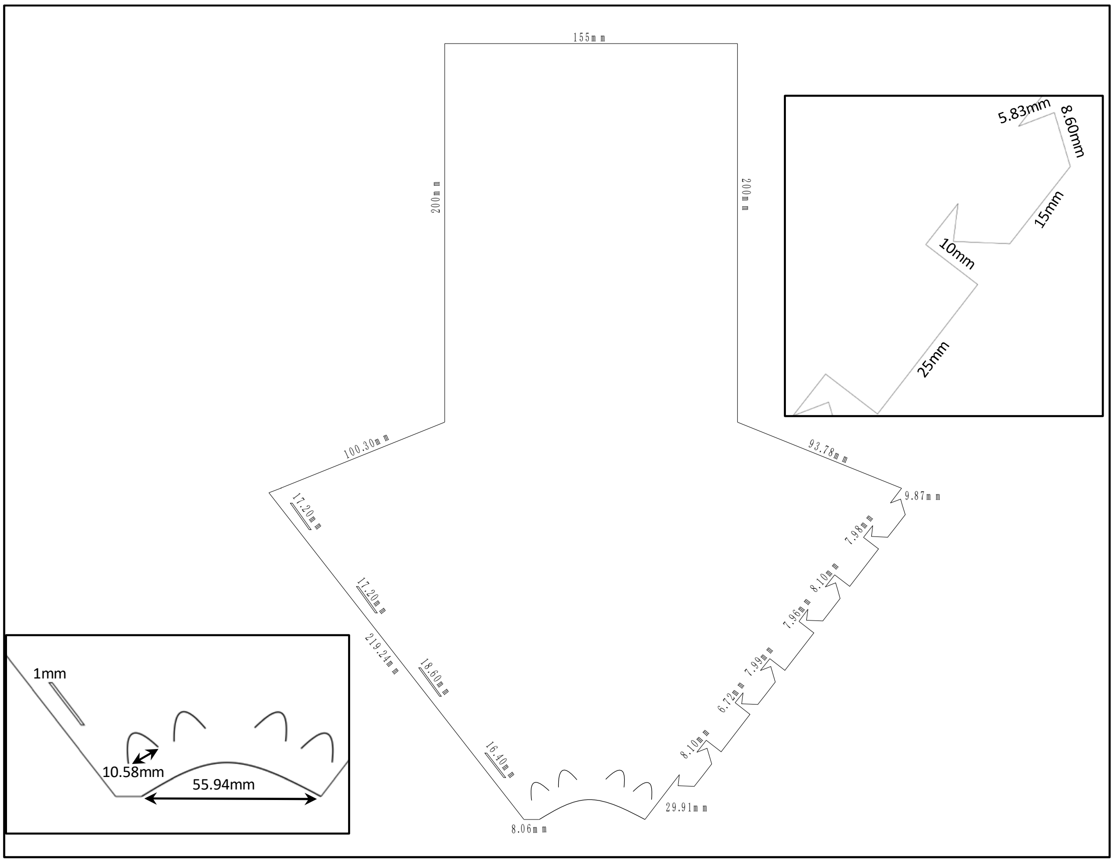

Figure S1. Detailed blueprint of a *Fan-trap* without fixation holes.
